# Dual function of fungi-derived cytokines in inflammatory bowel diseases: protection or inflammation

**DOI:** 10.1093/gastro/goad068

**Published:** 2023-12-05

**Authors:** Niusha Sharifinejad, Elaheh Mahmoudi

**Affiliations:** Non-Communicable Diseases Research Center, Alborz University of Medical Sciences, Karaj, Iran; Department of Mycology, School of Medicine, Alborz University of Medical Sciences, Karaj, Iran

**Keywords:** inflammatory bowel diseases, IBD, cytokines, intestinal fungi, fungi-derived cytokines

## Abstract

Inflammatory bowel disease (IBD) is an immune-mediated inflammatory condition involving both the innate and adaptive immune systems. Recently, the role of intestinal fungal flora and their downstream immune pathways has been highlighted in the pathogenesis of IBD. Cytokines as primary immune mediators require a delicate balance for maintaining intestinal homeostasis. Although most cytokines have a predictable role in either amplifying or attenuating inflammation in IBD, a few cytokines have shown a dual function in the inflammatory state of the intestine. Some of these dual-faced cytokines are also involved in mucosal anti-microbial defense pathways, particularly against intestinal fungal residents. Here, we reviewed the role of these cytokines in IBD pathogenesis to achieve a better understanding of the fungal interactions in the development of IBD.

## Introduction

Inflammatory bowel disease (IBD), which mainly includes Crohn’s disease (CD) and ulcerative colitis (UC), is a chronic multifactorial disorder that places a considerable burden on the global health system [1, 2]. IBD is generally defined as an excessive inflammatory response to intestinal microbiota in a genetically susceptible individual [3]. Fungi, as the second most prevalent residents of the intestine, are described to interfere with IBD pathogenesis [4]. An increased risk of IBD is observed in the presence of distinct genetic variations in fungal recognition receptors, leading to aberrant immune responses against fungal species and further cytokine production [5].

Cytokines govern key cellular processes in the intestine including cell death, proliferation, and inflammatory responses to pathogens. They constantly mediate the crosstalk between the immune and the epithelial cells even under homeostatic conditions [6]. IBD, as an immune-mediated inflammatory condition, alters the expression of a substantial number of cytokines [7, 8]. The altered cytokine network could result in either susceptibility or resistance to IBD. Therefore, recent biological therapies have targeted particular cytokines for better disease control; however, some of these potent biologic agents failed to induce remission [9]. Despite cytokines with a predictable role in either amplifying or attenuating inflammation of IBD, a few cytokines showed a dual function in the inflammatory state of the intestine [10]. The effect of these cytokines on IBD seems to be time- and dose-dependent, requiring a delicate intestinal homeostasis [11].

On the other hand, some of these dual-faced cytokines are also involved in mucosal anti-microbial defense against fungal pathogens [12]. Commensal gut fungi mainly act via the Dectin–spleen tyrosine kinase–caspase recruitment domain 9 signaling pathway and produce a wide range of cytokines [13]. These interactions result in protection or tolerance against fungal species and strengthen the role of an imbalanced intestinal fungal community in the development of IBD. Some studies have even indicated that distinct fungal species could initiate inflammatory or anti-inflammatory cascades in IBD [4, 14]. These specific cytokines include interleukin (IL)-1, IL-4, IL-6, IL-10, IL-13, IL-17, IL-18, IL-22, IL-23, IL-33, IL-35, tumor necrosis factor-alpha (TNF-α), and interferon-gamma (IFN-γ) [10, 11, 15, 16]. Since the mechanisms by which intestinal fungi affect IBD are not clear, we aimed to review these cytokines to achieve a better understanding of their role in IBD development and their probable interactions against intestinal fungi.

## Cytokine network in IBD

**Figure 1.**
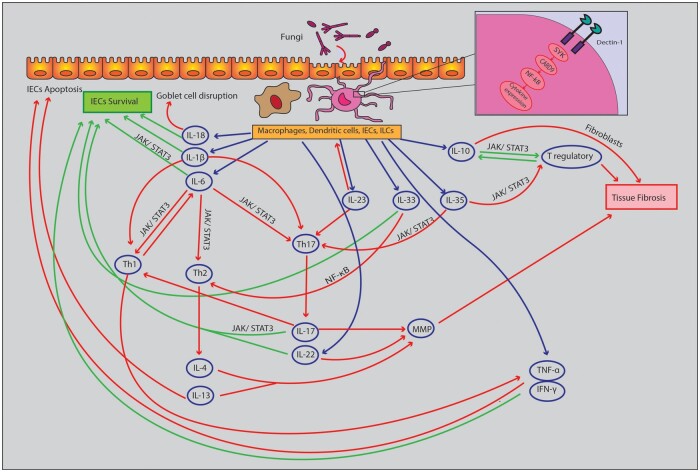
The cytokine network in pathogenesis of inflammatory bowel disease. These signaling pathways could be driven by intestinal fungal residents, leading to the maintenance of barrier integrity through increased IEC survival or mucosal injury and tissue fibrosis. Dectin-1, as the main receptor of fungi, induces cytokine expression via the SYK–CARD9 pathway. Red arrows and green arrows represent pro-inflammatory and anti-inflammatory pathways, respectively. CARD9 = caspase recruitment domain 9, IECs = intestinal epithelial cells, IFN-γ = interferon-gamma, IL = interleukin, ILC = innate lymphoid cells, JAK/STAT = Janus kinase/signal transducers and activators of transcription, MMP = matrix metalloproteinases, NF-κB = nuclear factor kappa B, SYK = spleen tyrosine kinase, Th = T helper, TNF-α = tumor necrosis factor-alpha.

In an inflamed intestinal mucosa, IL-1, IL-18, IL-6, and IL-23 are produced by intestinal epithelial cells, innate lymphoid cells, and phagocytes. Of these cytokines, IL-1, IL-18, and IL-6 promote the survival and proliferation of intestinal epithelial cells, thereby maintaining the intestinal barrier [10]. Simultaneously, in the opposite direction, IL-1β induces T helper (Th) 1 and Th17 differentiation [17] and IL-18 disrupts goblet cell maturation and function [18]. In colitis models, both IL-1β and IL-18 generally drive intestinal inflammation [19, 20]. IL-6 signaling also prevents apoptosis and promotes the survival of Th1, Th2, or Th17 CD4+ T cells through activating signal transducers and the activator of the transcription 3 (STAT3) signaling pathway, which explains the contrasting effects of IL-6 [21]. Thus, the pro-inflammatory effects arise from increasing T-cell survival and the anti-inflammatory effects from maintaining intestinal epithelial cells [22]. Additionally, IL-23 is involved in the expansion and survival of Th17, natural killer cells, and innate lymphoid cells [23]. Figure 1 provides a better understanding of the cytokine signaling pathways in IBD.

Upon stimulation, IL-10 is produced primarily by dendritic cells and lymphocytes, especially regulatory T cells [24]. After binding to its heterotetrameric receptor, IL-10 initiates an immune cascade to phosphorylate STAT3 and enhances immune tolerance through balancing polymorphonuclear leukocyte-regulatory T-cell interaction [25]. IL-10/IL-10R signaling defects are a known underlying etiology for early-onset IBD in pediatric patients [26]. Despite the well-known immunomodulatory effects of IL-10, its overexpression could lead to tissue fibrosis by activating fibroblasts [15, 27].

IL-33 and IL-35 are other innate immune-associated cytokines, primarily derived from intestinal epithelial cells, sub-epithelial myofibroblasts, and dendritic cells. IL-33 and Toll-like receptors commonly share the MyD88-dependent pathway, which leads to nuclear factor kappa B (NF-κB) and mitogen-activated protein kinase activation. Subsequently, a set of pro-inflammatory cytokines is expressed, disrupting the tolerance against intestinal pathogens [28]. Additionally, IL-33 promotes Type 2 immune responses and could act as an alarm in response to cellular stress, to induce intestinal epithelial cell proliferation and repair [28]. IL-35 is a member of the IL-12 cytokine family and consists of IL-12p35 and IL-27b subunits that exert function through activating Janus tyrosine kinase/STAT3 signaling. While the IL-12p35 subunit demonstrates a suppressive role in inflammatory/autoimmune conditions [29], IL-27b is highly expressed on regulatory T cells and Th17 cells, contributing to immunomodulation [30]; IL-35 is associated with colitis exacerbation [31].

Following the activation of Th17, cytokines such as IL-17 (commonly IL-17A and IL-17F) and IL-22 are released [32]. In addition to Th17, innate lymphoid cells, Th1, and natural killer T cells are the other sources of IL-22 expression [6]. IL-17A, IL-17F, and IL-22 act on Th1 and intestinal fibroblasts to secrete pro-inflammatory mediators and matrix metalloproteinases, respectively, leading to either intestinal inflammation or fibrosis [33–36]. Furthermore, IL-17 and IL-22 along with IL-6 enhance anti-microbial peptide secretion and intestinal epithelial cell permeability and proliferation via STAT3 phosphorylation [37–39]. In turn, STAT3 downregulates inflammatory mediators and maintains intestinal barrier integrity [24, 37].

Activated Th2 cells induce IL-4 and IL-13 cytokines; IL-13 is released from natural killer T cells as well [40]. IL-13 and IL-4 can prompt intestinal fibrosis by down-regulating matrix metalloproteinase synthesis in fibroblasts, which has led to collagen accumulation in the intestinal tissue of *in vitro* and *in vivo* experimental models of CD and colitis [41, 42]. This effect promotes wound healing and reduces inflammation in the reparatory phase of intestinal inflammation. However, unresolved inflammation results in chronic inflammation, uncontrolled tissue remodeling, and ultimately tissue fibrosis [43]. Furthermore, IL-13, but not IL-4, can impair epithelial barrier function by inducing epithelial apoptosis, and altering tight junctions and restitution velocity in a dose-dependent manner [44]. Previous studies have demonstrated an inhibitory effect of IL-4 and IL-13 on inflammation in IBD [10]. However, some data represented their potential roles in IBD pathogenesis. Despite the clear fibrotic mechanisms of IL-4 in skin and hepatic tissue [45, 46], its potential role in intestinal fibrosis is still controversial [47]. IL-4 was able to perpetuate or resolve inflammation in IBD studies [48].

Upon Th1 activation, a range of inflammatory cytokines are secreted: IL-6, TNF-α, and IFN-γ. TNF-α and IFN-γ are also released by innate immune cells such as macrophages [11], then TNF-α facilitates various biological activities through its Type 1 and Type 2 receptors. In murine intestinal models, excessive TNF-α produced by innate immune components weakens barrier function and suppresses T-cell apoptosis [22, 49]. Conversely, TNF-α contributes to homeostatic bioactivities after binding to TNF receptor 2 and promotes tissue regeneration, cell proliferation, and survival [50]. Despite the established pro-inflammatory role of IFN-γ and promising inhibitory effects of anti-IFN-γ in IBD [11, 51], a recent article illustrated the protective effects of IFN-γ in T-cell-mediated colitis [52].

Interestingly, CD is usually designated as a Type 1-driven disease with an elevated activation of Th1 and Th17 cells and their related cytokines, whereas UC is more associated with Type 2 inflammation (Th2 cell activation) and subsequent cytokine production [53].

## Cytokines and fungal pathogens

**Figure 2.**
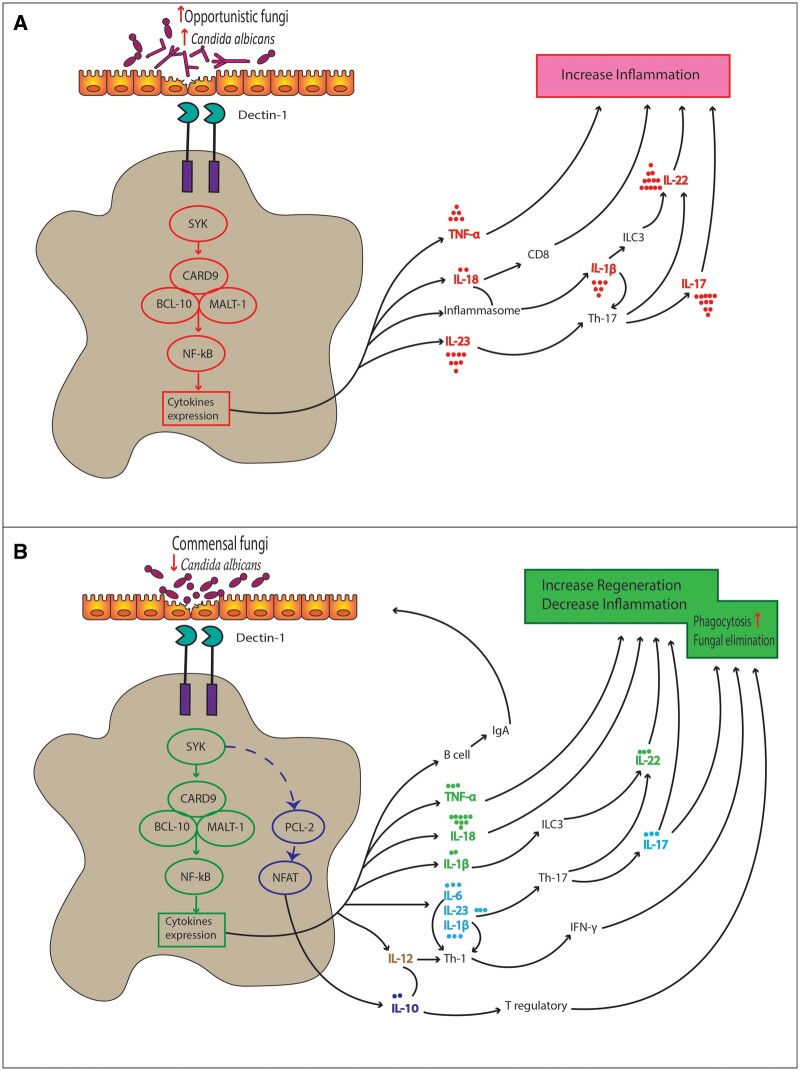
Negative effects of mucosal immunity and cytokine induction in response to opportunistic fungi (A) and positive effects of cytokines in response to commensal fungi (B). In addition to the type of expressed cytokines, the dose of induction determines the type of immune responses to different fungal flora. Opportunistic fungi upregulate IL-1β, IL-17, IL-22, IL-23, and TNF-α production, leading to mucosal inflammation, whereas these cytokines are downregulated in response to commensal fungi, leading to regeneration. The level of expression of various cytokines is displayed with a dot in the same color. BCL-10 = B-cell lymphoma 10, CARD9 = caspase recruitment domain 9, IFN-γ = interferon-gamma, Ig = immunoglobulin, IL = interleukin, ILC = innate lymphoid cells, MALT-1 = mucosa-associated lymphoid tissue lymphoma translocation protein 1, NFAT = nuclear factor of activated T cells, NF-κB = nuclear factor kappa B, SYK = spleen tyrosine kinase, Th = T helper, TNF-α = tumor necrosis factor-alpha.

Recent studies have proposed tissue-resident fungi as immune modulators contributing to the pathogenesis of autoimmune, inflammatory, and neoplastic diseases [54]. At the intestinal mucosal surface, C-type lectin receptors interact with fungal cell wall components and initiate a signal via spleen tyrosine kinase, a caspase recruitment domain 9, B-cell lymphoma 10, and mucosa-associated lymphoid tissue lymphoma translocation protein 1 complex, and/or Raf-1 proto-oncogene, serine-threonine kinase. These signaling pathways activate the production of IL-23, IL-6, IL-10, IL-2, IL-1, and TNF-α by phagocytes (macrophages, monocytes, dendritic cells, and neutrophils) [55]. The interaction with fungi-induced phagocytes results in the development of Th1 and Th17 cells, and recruitment of neutrophils to the intestinal lamina propria [3]. Commensal fungi play an important role in inflammasome activation via the spleen tyrosine kinase–caspase recruitment domain 9 pathway and its downstream IL-18, which is required for IFN-γ release from T cells. This signaling pathway of commensal gut fungi could promote anti-tumorigenic T-cell responses and eventually protect against colitis and colon cancer [13]. IL-1β and IL-18 generally represent similar roles in mucosal immunity against fungi after the stimulation of Dectin-1 [56, 57]. Furthermore, IL-1β modulates Group 3 innate lymphoid cells to produce IL-22 [13]. In response to intestinal fungi and mucosal fungal infection, IL-6 amplifies and eliminates the pathogens via recruiting neutrophils and augmenting Th1-mediated immunity [58–60]. *Malassezia restricta* is particularly potent at inducing IL-6 expression from mouse dendritic cells [14]. The detailed signaling pathway of commensal gut fungi is presented in Figure 2.

Moreover, the contribution of IL-10 to antifungal responses is associated with conflicting outcomes. IL-10 inhibits the fungicidal activity of monocytes or neutrophils [61–63], whereas it is required for the optimal development of IL-12-dependent Th1 cells and regulatory T cells [64, 65]. Recently a novel probiotic yeast attenuated colitis by upregulating IL-10 in mice [66]. An *in vitro* study of murine dendritic cells showed a significant elevation of IL-10 following stimulation with *Saccharomyces cerevisiae*, a species decreased in IBD patients [4], suggesting an association between specific fungal strains and distinct cytokines.

IL-33 has an established role in enhancing resistance and tolerance to fungal pathogens in animal models [67, 68]. IL-33 administration limits fungal burden by increasing neutrophil phagocytic activity. Conversely, IL-33 stimulates Th2 cells to produce IL-13, which, in turn, drives macrophages towards the M2 subtype and subsequent immunosuppression [69]. Moreover, IL-33 mediated regulatory T-cell differentiation and restricted the IL-23 signaling pathway in a bacteria-driven colitis model [70]. Additionally, IL-35 exerts a p35-dependent antifungal response downstream of IL-22 [71]. Furthermore, *IL12p35*^–/–^ mice had a lower fungal burden during oral candidiasis, indicating the participation of IL-35 in reducing tissue damage [72], while *Candida albicans* raised the IL-27b level and suppressed inflammation [73].

In a similar way, the IL-23/Th17 signaling pathway has a controversial role in tolerance to fungal flora by acting on both regulatory T- and Th1-cell responses [73]. Dectin-1 is the main fungal receptor that initiates a cascade leading to Th17 activation, followed by IL-17 and IL-22 production [74]. IL-17 is involved in both response and resistance to fungal species. It promotes neutrophil recruitment and natural killer cell trafficking in murine models [75, 76]. Additionally, a former study reported increased levels of mucosal IL-23 and IL-17A in UC patients and their correlation with specific fungal genera in the intestine [60]. IL-22 has a more crucial role than IL-17 in the mucosal host defense against *C. albicans*. In animal models, IL-22-deficient mice were highly susceptible to intestinal candidiasis [71].

The Th2 pathway and its mediators, including IL-4 and IL-13, are also involved in antifungal immunity (Table 1). IL-4 was found to suppress protective immune responses to *Aspergillus fumigatus* and *Histoplasma capsulatum* [77, 78]; IL-13 induced tolerance to *Cryptococcus neoformans* in murine models [79]. Aside from the expected resistance of both IL-4 and IL-13 against fungal species [12], IL-4 takes part in the optimal development of IL-12-dependent Th1 responses [80] and IL-13 increases oxidative burst and phagocytosis during fungal infections [79, 81]. Similarly to IBD, these cytokines have contrary effects against fungal pathogens that are generally time- and dose-dependent [82]; therefore, a regulated balance is required to maintain intestinal homeostasis during inflammation. No previous study has examined the relevance of IL-4 and IL-13 to intestinal fungi in IBD; however, Th2-related cytokines are involved in mucosal immunity against gut fungi [59].

**Table 1.** Selected cytokine activities implicated in the pathogenesis of IBD and fungal immunity

Cytokine	Source in the mucosa	Potential function in the pathogenesis of intestinal inflammation	Potential function against mucosal fungi
IL-1	Phagocytes, ILCs, and IECs	Promote Th1 and Th17 differentiation, and IFN-γ secretion; increase the survival of IECs	Promote Th1 and Th17 differentiation, and IFN-γ secretion; modulate Group 3 ILCs to produce IL-22
IL-4	T cells	Intestinal fibrosis; tissue remodeling	Induce IL-12-dependent Th1 responses; suppress protective immune responses to *Aspergillus fumigatus* and *Histoplasma capsulatum*
IL-6	Phagocytes, ILCs, and IECs	Increase the survival of IECs, Th1, Th2, or Th17 CD4+ T cells; increase the survival of IECs and anti-microbial peptide secretion	Neutrophil recruitment; augmenting Th1-mediated immunity
IL-10	Dendritic cells and T cells	Balancing polymorphonuclear leukocyte-regulatory T-cell interaction; tissue fibrosis	Inhibit the fungicidal activity of monocytes and neutrophils; induce IL-12-dependent Th1 cells and Treg cells
IL-13	T cells	Intestinal fibrosis; impair epithelial barrier	Oxidative burst; phagocytosis; induce tolerance to *Cryptococcus neoformans*
IL-17	T cells (Th17)	Intestinal fibrosis; recruit neutrophils; anti-microbial peptide secretion; intestinal barrier survival	Neutrophil recruitment; natural killer cells trafficking; upregulate Treg and Th1 responses
IL-18	Phagocytes, ILCs, and IECs	Disrupt goblet cells maturation and function; increase the survival of IECs; induce anti-tumorogenic T-cell responses	Induce anti-tumorogenic T-cell responses
IL-22	T cells (Th17, ILCs, Th1, and natural killer T cells)	Intestinal barrier repair; tissue fibrosis; mucosal injury; anti-microbial peptide secretion	Upregulate Treg and Th1 responses; induce inflammation in response to intestinal *Candida albicans*
IL-23	Phagocytes, ILCs, and IECs	Expansion and survival of Th17, natural killer cells, and ILCs	Promote Th1 and Th17 differentiation; upstream of IL-17 and IL-22
IL-33	IECs and sub-epithelial myofibroblasts	Induce inflammation during early stages of acute colitis; generate a shift towards Th2 immune reactions; increase the survival of IECs	Increase neutrophil phagocytic activity; shift immune responses toward M2 macrophages; induce Treg differentiation
IL-35	Dendritic cells	Upregulate Treg cells and Th17	Upregulate Treg cells; suppress inflammation; p35-dependent antifungal response downstream of IL-22
TNF-α	Innate immune components; T cells	Weaken barrier function; suppress T-cell apoptosis; neutrophil recruitment	Neutrophil recruitment; increase T-cell survival
IFN-γ	Innate immune components; T cells	Suppress T-cell apoptosis; neutrophil recruitment	Neutrophil recruitment

IBD = inflammatory bowel disease, IECs = intestinal epithelial cells, IFN-γ = interferon-gamma, IL = interleukin, ILCs = innate immune cells, Th = T helper, TNF-α = tumor necrosis factor-alpha, Treg = T regulatory.

Intriguingly, the production of TNF-α and IFN-γ was strikingly increased in the inflamed mucosa of both CD and UC patients, in which intestinal mycobiome richness and diversity were positively correlated with TNF-α and IFN-γ expression [16, 59]. Further analysis of cytokine expression demonstrated that commensal fungi such as *C. albicans* upregulated the production of IL-2, IL-10, IL-17, TNF-α, and IFN-γ, restricted to the fungus-specific memory T cells [83].

## Cytokine-targeted therapies in IBD

### Interleukin-4 and interleukin-13

Anrukinzumab, an anti-IL-13 antibody that impedes the binding of IL-13 and IL-4Rα, and tralokinumab (IL-13 neutralizing antibody) failed to significantly improve the clinical condition of patients with moderate-to-severe UC [84, 85]. However, promising effects of IL-4/IL-13 dual antagonist were observed on murine colitis models [86]. Exogenous IL-4 treatment had controversial effects on clinical remission and mucosal healing in colitis models [87, 88].

### Interleukin-23, interleukin-17, and interleukin-22

Ustekinumab is an anti-IL-12/IL-23 agent that is approved for the treatment of both CD and UC; it binds the shared p40 subunit of cytokines IL-12 and IL-23, and prevents the cytokine from joining to its receptor [89]. However, the favorable outcomes may stem from antagonizing IL-12, which is one of the primary mediators of Th1-induced immunity. The clinical efficacy of selective IL-23/p19 inhibitors (such as mairikizumab, risankizumab, brazikumab, and guselkumab) is still an ongoing project that needs to be clarified in further clinical trials [90].

IL-17A and IL-17F functions seem to be determined by the target organ. Unlike skin, IL-17A is pivotal for intestinal barrier survival [91, 92], which does not explain the ineffectiveness of anti-IL-17A therapy (secukinumab) in moderate-to-severe CD [93]. IL-17 inhibitors even exacerbated intestinal inflammation [94]. In a retrospective study of IBD patients, IL-17 inhibitors including secukinumab, ixekizumab, and brodalumab were associated with exacerbation and new onset of IBD and colitis [95]. On the other hand, although IL-22 administration resulted in more severe mucosal injury in colitis models [96], prophylactic treatment with IL-22 in *Citrobacter rodentium*-induced colitis models ameliorated the intestinal epithelial dysfunction [97]. There are ongoing clinical trials regarding the effect of IL-22Fc fusion protein (Efmarodocokin alfa, UTTR1147A) in IBD patients (NCT02749630). Table 2 provides a summary of cytokine-based therapeutic clinical trials in IBD.

**Table 2.** The summary of IBD clinical trials targeting the selected cytokines

Cytokine	Clinical trial result
IL-1	IL-1R antagonist (anakinra) could not reduce the need for rescue therapy or colectomy in patients with acute severe UC
IL-6	PF-04236921 (anti-IL-6 antibody), tocilizumab, and olamkicept (anti-IL-6R) showed plausible results in IBD patients
IL-10	Recombinant IL-10 therapies revealed limited or no efficacy in IBD remission
IL-13	Anrukinzumab and tralokinumab (IL-13 neutralizing antibody) failed to significantly improve the clinical condition of patients with moderate-to-severe UC
IL-17	Anti-IL-17A antibodies (secukinumab, ixekizumab, and brodalumab) were ineffective for IBD
IL-23	Ustekinumab is the only anti-IL-12/IL-23 agent approved for the treatment of both CD and UC
TNF-α	Anti-TNF-α agents (infliximab, adalimumab, golimumab, AVX-470, and certolizumab pegol) are the main biologic therapies in IBD
IFN-γ	Fontolizumab (anti-IFN-γ antibody) increased the rate of clinical response in individuals with refractory CD

IL = interleukin, TNF-α = tumor necrosis factor-alpha, IBD = inflammatory bowel disease, UC = ulcerative colitis, CD = Crohn’s disease, R = receptor.

### Interleukin-10

Recombinant IL-10 therapies have been tested in multiple IBD clinical trials but revealed limited or no efficacy in disease remission [98, 99].

### Interleukin-1 and interleukin-18

Blockade of IL-1β and IL-18 reduced intestinal inflammation in murine models of UC [100, 101]. However, no clinical trial has ever investigated the blockade of IL-1β and IL-18 in IBD patients. Additionally, anakinra (IL-1R antagonist) could not reduce the need for rescue therapy or colectomy in patients with acute severe UC [102].

### Interleukin-6

Different anti-IL-6/IL-6R therapeutic options have shown plausible results in IBD clinical trials [103, 104]. PF-04236921 is an anti-IL-6 antibody that induced clinical remission in refractory CD patients who were unresponsive to anti-TNF regimens [103]. Tocilizumab and olamkicept are other prospective IL-6R inhibitors in IBD target therapies [104].

### Interleukin-33

Studies have shown that both IL-33 deficiency and the administration of recombinant IL-33 ameliorated experimental colitis in mice. In fact, treatment with IL-33 exacerbates the disease severity at the onset of dextran sodium sulfate-induced colitis, whereas it ameliorates the disease during the recovery phases [105].

### Interleukin-35

Recombinant IL-35 reversed the inflammatory indices and mucosal damage in murine models of active colitis [106].

### Interferon-gamma and tumor necrosis factor-alpha

Anti-TNF-α agents are the cornerstones of IBD biological treatments [107]. Four of them are widely used in the treatment of IBD: infliximab, adalimumab, golimumab, and certolizumab pegol. They generally bind to TNF-α and cause cell lysis of macrophages and T cells [9]. Intravenous administration of infliximab induces and maintains clinical remission and mucosal healing in patients with refractory or unresponsive IBD [108]. In contrast to infliximab and adalimumab, etanercept was associated with the development of paradoxical IBD, suggesting that other mechanisms beyond TNF-α neutralization are involved in the therapeutic effect of anti-TNF-α in IBD [109]. Additionally, AVX-470 is a new oral anti-TNF antibody that has improved the clinical, endoscopic, and inflammatory biomarkers for refractory UC with a good safety profile [110]. Moreover, unlike the intestinal bacterial community, anti-TNF therapy did not alter the gut fungal composition [111].

IFN-γ blockade in dextran sodium sulfate-colitis mouse models had increased angiogenesis accompanied with improved vascular barrier function and reduced colonic inflammation [112]. Fontolizumab, an anti-IFN-γ antibody, increased the rate of clinical response in individuals with refractory CD, after receiving two doses of fontolizumab intravenously on Day 56 [113].

## Discussion

Here, we reviewed the dual-faced cytokines involved in IBD pathogenesis as well as intestinal and mucosal antifungal responses. Despite a few controversial results, the cytokine network of both IBD and fungal-induced mucosal immunity had similar functions. Interestingly, distinct fungal species induced pro- or anti-inflammatory reactions during IBD, as *M. restricta* upregulated IL-6 [14] and *S. cerevisiae* induced IL-10 expression [4]. Dectin-1 played the vital role of the gatekeeper in initiating the immune responses against fungal flora. In general, the intestinal fungal composition seems to determine the type and the dose of cytokine expression and their role in the intestinal environment (Figure 2). Thus, an increase in opportunistic fungi such as *C. albicans* induced inflammation, whereas commensal fungi richness resulted in epithelial regeneration. However, no cytokine is specific for a distinct type of microorganism including bacteria, fungi, and viruses. It should be considered that, during intestinal inflammation, some bacteria and fungi may act via their inter-kingdom network rather than directly via the host [5]. Therefore, missing data regarding the probable confounding effect of other intestinal residents on fungal load need to be determined in future studies.

The biological therapies targeting the dual-faced cytokines have mainly failed to achieve remission in IBD clinical trials. In the reviewed literature, IFN-γ and IL-6 were the only cytokines with inflammatory effects against fungal species despite their dual function in IBD pathogenesis. On the other hand, anti-IL-6 and anti-IFN-γ antibodies such as olamkicept and fontolizumab significantly improved the clinical condition of IBD-affected patients. Thus, it could be speculated that treatments attenuating inflammatory responses against intestinal fungi are probably more effective. However, the scarce information regarding therapeutic options in the current study should be considered.

Most of our data were obtained from *in vitro* or animal models. Besides, considering the novelty of fungi to be regarded as the driving agent of cytokines and other limitations of the current study including lack of attention to the individual-specific composition of human intestinal fungi and insufficient sample size in a substantial number of reviewed studies, these conclusions should be regarded with caution. Of note, although the cytokines with a predictable role also work in the interaction of fungi with IBD pathogenesis, we reviewed the cytokines with dual functions to highlight the probable role of this association in IBD development.

## Conclusions

This review suggests that the intestinal fungal flora could probably contribute more to IBD genesis and drug resistance than we expected. We propose that the type of immune responses and cytokine expression in IBD might be influenced by the composition of intestinal fungal residents. Thus, altering the fungal flora in IBD patients may improve their clinical condition. However, further investigation should be followed by more clinical data and a larger sample size.

## References

[1] Jairath V , Feagan BG. Global burden of inflammatory bowel disease. Lancet Gastroenterol Hepatol 2020;5:2–3. 31648974 10.1016/S2468-1253(19)30358-9

[2] Venner JM , Bernstein CN. Immunomodulators: still having a role? Gastroenterol Rep (Oxf) 2022;10:goac061. 36381225 10.1093/gastro/goac061 PMC9642324

[3] Mahmoudi E , Mozhgani S-H, Sharifinejad N. The role of mycobiota-genotype association in inflammatory bowel diseases: a narrative review. Gut Pathog 2021;13:31. 33964975 10.1186/s13099-021-00426-4 PMC8106830

[4] Sokol H , Leducq V, Aschard H et al Fungal microbiota dysbiosis in IBD. Gut 2017;66:1039–48. 26843508 10.1136/gutjnl-2015-310746 PMC5532459

[5] Underhill DM , Braun J. Fungal microbiome in inflammatory bowel disease: a critical assessment. J Clin Invest 2022;132. 10.1172/JCI155786 PMC8884899 35229726

[6] Mahapatro M , Erkert L, Becker C. Cytokine-mediated crosstalk between immune cells and epithelial cells in the gut. Cells 2021;10. 10.3390/cells10010111 PMC7827439 33435303

[7] Geremia A , Biancheri P, Allan P et al Innate and adaptive immunity in inflammatory bowel disease. Autoimmun Rev 2014;13:3–10. 23774107 10.1016/j.autrev.2013.06.004

[8] Racke MK , Bonomo A, Scott DE et al Cytokine-induced immune deviation as a therapy for inflammatory autoimmune disease. J Exp Med 1994;180:1961–6. 7525845 10.1084/jem.180.5.1961 PMC2191757

[9] Moreno LO , Fernández-Tomé S, Abalo R. Biological treatments in inflammatory bowel disease: a complex mix of mechanisms and actions. Biologics 2021;1:189–210.

[10] Neurath MF. Cytokines in inflammatory bowel disease. Nat Rev Immunol 2014;14:329–42. 24751956 10.1038/nri3661

[11] Friedrich M , Pohin M, Powrie F. Cytokine networks in the pathophysiology of inflammatory bowel disease. Immunity 2019;50:992–1006. 30995511 10.1016/j.immuni.2019.03.017

[12] Romani L. Immunity to fungal infections. Nat Rev Immunol 2004;4:1–23. 14661066 10.1038/nri1255

[13] Malik A , Sharma D, Malireddi RKS et al SYK-CARD9 signaling axis promotes gut fungi-mediated inflammasome activation to restrict colitis and colon cancer. Immunity 2018;49:515–30.e5. 30231985 10.1016/j.immuni.2018.08.024 PMC6541497

[14] Limon JJ , Tang J, Li D et al Malassezia is associated with Crohn’s disease and exacerbates colitis in mouse models. Cell Host Microbe 2019;25:377–88.e6. 30850233 10.1016/j.chom.2019.01.007 PMC6417942

[15] Steen EH , Wang X, Balaji S et al The role of the anti-inflammatory cytokine interleukin-10 in tissue fibrosis. Adv Wound Care (New Rochelle) 2020;9:184–98. 32117582 10.1089/wound.2019.1032 PMC7047112

[16] Li Q , Wang C, Tang C et al Dysbiosis of gut fungal microbiota is associated with mucosal inflammation in Crohn’s disease. J Clin Gastroenterol 2014;48:513–23. 24275714 10.1097/MCG.0000000000000035 PMC4059552

[17] Coccia M , Harrison OJ, Schiering C et al IL-1β mediates chronic intestinal inflammation by promoting the accumulation of IL-17A secreting innate lymphoid cells and CD4+ Th17 cells. J Exp Med 2012;209:1595–609. 22891275 10.1084/jem.20111453 PMC3428945

[18] Nowarski R , Jackson R, Gagliani N et al Epithelial IL-18 equilibrium controls barrier function in colitis. Cell 2015;163:1444–56. 26638073 10.1016/j.cell.2015.10.072 PMC4943028

[19] Lopetuso LR , Chowdhry S, Pizarro TT. Opposing functions of classic and novel IL-1 family members in gut health and disease. Front Immunol 2013;4:181. 23847622 10.3389/fimmu.2013.00181 PMC3705591

[20] Dinarello CA , Novick D, Kim S et al Interleukin-18 and IL-18 binding protein. Front Immunol 2013;4:289. 24115947 10.3389/fimmu.2013.00289 PMC3792554

[21] Hunter CA , Jones SA. IL-6 as a keystone cytokine in health and disease. Nat Immunol 2015;16:448–57. 25898198 10.1038/ni.3153

[22] Atreya R , Mudter J, Finotto S et al Blockade of interleukin 6 trans signaling suppresses T-cell resistance against apoptosis in chronic intestinal inflammation: evidence in Crohn disease and experimental colitis in vivo. Nat Med 2000;6:583–8. 10802717 10.1038/75068

[23] Gutcher I , Becher B. APC-derived cytokines and T cell polarization in autoimmune inflammation. J Clin Invest 2007;117:1119–27. 17476341 10.1172/JCI31720 PMC1857272

[24] Iyer SS , Cheng G. Role of interleukin 10 transcriptional regulation in inflammation and autoimmune disease. Crit Rev Immunol 2012;32:23–63. 22428854 10.1615/critrevimmunol.v32.i1.30 PMC3410706

[25] Maynard CL , Harrington LE, Janowski KM et al Regulatory T cells expressing interleukin 10 develop from Foxp3+ and Foxp3- precursor cells in the absence of interleukin 10. Nat Immunol 2007;8:931–41. 17694059 10.1038/ni1504

[26] Zhu L , Shi T, Zhong C et al IL-10 and IL-10 receptor mutations in very early onset inflammatory bowel disease. Gastroenterology Res 2017;10:65–9. 28496525 10.14740/gr740w PMC5412537

[27] Braat H , Peppelenbosch MP, Hommes DW. Interleukin-10-based therapy for inflammatory bowel disease. Expert Opin Biol Ther 2003;3:725–31. 12880373 10.1517/14712598.3.5.725

[28] Aggeletopoulou I , Tsounis EP, Triantos C. Molecular mechanisms underlying IL-33-Mediated inflammation in inflammatory bowel disease. Int J Mol Sci 2022;24:623. 36614065 10.3390/ijms24010623 PMC9820409

[29] Su L-C , Liu X-Y, Huang A-F et al Emerging role of IL-35 in inflammatory autoimmune diseases. Autoimmun Rev 2018;17:665–73. 29729445 10.1016/j.autrev.2018.01.017

[30] Collison LW , Workman CJ, Kuo TT et al The inhibitory cytokine IL-35 contributes to regulatory T-cell function. Nature 2007;450:566–9. 18033300 10.1038/nature06306

[31] Wirtz S , Billmeier U, Mchedlidze T et al Interleukin-35 mediates mucosal immune responses that protect against T-cell–dependent colitis. Gastroenterology 2011;141:1875–86. 21820391 10.1053/j.gastro.2011.07.040 PMC3624892

[32] Kobayashi T , Okamoto S, Hisamatsu T et al IL23 differentially regulates the Th1/Th17 balance in ulcerative colitis and Crohn’s disease. Gut 2008;57:1682–9. 18653729 10.1136/gut.2007.135053

[33] Leppkes M , Becker C, Ivanov II et al RORgamma-expressing Th17 cells induce murine chronic intestinal inflammation via redundant effects of IL-17A and IL-17F. Gastroenterology 2009;136:257–67. 18992745 10.1053/j.gastro.2008.10.018

[34] Jin W , Dong C. IL-17 cytokines in immunity and inflammation. Emerg Microbes Infect 2013;2:e60–e60. 26038490 10.1038/emi.2013.58 PMC3820987

[35] Curciarello R , Docena G, Macdonald T. The role of cytokines in the fibrotic responses in Crohn’s disease. Front Med (Lausanne) 2017;4:126. 28824915 10.3389/fmed.2017.00126 PMC5545939

[36] Fuss IJ . IL-17: intestinal effector or protector? Mucosal Immunol 2011;4:366–7.

[37] Pickert G , Neufert C, Leppkes M et al STAT3 links IL-22 signaling in intestinal epithelial cells to mucosal wound healing. J Exp Med 2009;206:1465–72. 19564350 10.1084/jem.20082683 PMC2715097

[38] Kuhn KA , Schulz HM, Regner EH et al Bacteroidales recruit IL-6-producing intraepithelial lymphocytes in the colon to promote barrier integrity. Mucosal Immunol 2018;11:357–68. 28812548 10.1038/mi.2017.55 PMC5815964

[39] Chaudhry A , Rudra D, Treuting P et al CD4+ regulatory T cells control TH17 responses in a stat3-dependent manner. Science 2009;326:986–91. 19797626 10.1126/science.1172702 PMC4408196

[40] Junttila IS. Tuning the cytokine responses: an update on interleukin (IL)-4 and IL-13 receptor complexes. Front Immunol 2018;9:888. 29930549 10.3389/fimmu.2018.00888 PMC6001902

[41] Bailey JR , Bland PW, Tarlton JF et al IL-13 promotes collagen accumulation in crohn’s disease fibrosis by down-regulation of fibroblast MMP synthesis: a role for innate lymphoid cells? PLoS One 2012;7:e52332. 23300643 10.1371/journal.pone.0052332 PMC3534115

[42] Fichtner-Feigl S , Young CA, Kitani A et al IL-13 signaling via IL-13R alpha2 induces major downstream fibrogenic factors mediating fibrosis in chronic TNBS colitis. Gastroenterology 2008;135:2003–13. 2013.e1-7. 18938165 10.1053/j.gastro.2008.08.055

[43] Karin M , Clevers H. Reparative inflammation takes charge of tissue regeneration. Nature 2016;529:307–15. 26791721 10.1038/nature17039 PMC5228603

[44] Heller F , Florian P, Bojarski C et al Interleukin-13 is the key effector Th2 cytokine in ulcerative colitis that affects epithelial tight junctions, apoptosis, and cell restitution. Gastroenterology 2005;129:550–64. 16083712 10.1016/j.gastro.2005.05.002

[45] Aoudjehane L , Pissaia A, Scatton O et al Interleukin-4 induces the activation and collagen production of cultured human intrahepatic fibroblasts via the STAT-6 pathway. Lab Invest 2008;88:973–85. 18626468 10.1038/labinvest.2008.61

[46] Postlethwaite AE , Holness MA, Katai H et al Human fibroblasts synthesize elevated levels of extracellular matrix proteins in response to interleukin 4. J Clin Invest 1992;90:1479–85. 1401080 10.1172/JCI116015 PMC443194

[47] Yang B , Zhang G, Elias M et al The role of cytokine and immune responses in intestinal fibrosis. J Dig Dis 2020;21:308–14. 32410365 10.1111/1751-2980.12879

[48] Imam T , Park S, Kaplan MH et al Effector T helper cell subsets in inflammatory bowel diseases. Front Immunol 2018;9:1212. 29910812 10.3389/fimmu.2018.01212 PMC5992276

[49] Pott J , Kabat AM, Maloy KJ. Intestinal epithelial cell autophagy is required to protect against TNF-induced apoptosis during chronic colitis in Mice. Cell Host Microbe 2018;23:191–202.e4. 29358084 10.1016/j.chom.2017.12.017

[50] Jang DI , Lee AH, Shin HT et al The role of tumor necrosis factor alpha (TNF-α) in autoimmune disease and current tnf-α inhibitors in therapeutics. Int J Mol Sci 2021;22:2719. 33800290 10.3390/ijms22052719 PMC7962638

[51] Ghosh S , Chaudhary R, Carpani M et al Interfering with interferons in inflammatory bowel disease. Gut 2006;55:1071–3. 16849343 10.1136/gut.2005.090134 PMC1856258

[52] Zheng SG , Xu Z, Wang J. A protective role of IFN-γ in T cell-mediated colitis by regulation of Treg/Th17 via induction of indoleamine-2,3-deoxygenase. J Immunol 2019;202:57.3–.3.

[53] Gomez-Bris R , Saez A, Herrero-Fernandez B et al CD4 T-Cell Subsets and the Pathophysiology of Inflammatory Bowel Disease. Int J Mol Sci 2023;24:2696. 36769019 10.3390/ijms24032696 PMC9916759

[54] Lionakis MS , Drummond RA, Hohl TM. Immune responses to human fungal pathogens and therapeutic prospects. Nat Rev Immunol 2023;23:433–52. 36600071 10.1038/s41577-022-00826-w PMC9812358

[55] Speakman EA , Dambuza IM, Salazar F et al T Cell antifungal immunity and the role of c-type lectin receptors. Trends Immunol 2020;41:61–76. 31813764 10.1016/j.it.2019.11.007 PMC7427322

[56] Yuan K , Zhao G, Che C et al Dectin-1 is essential for IL-1β production through JNK activation and apoptosis in *Aspergillus fumigatus* keratitis. Int Immunopharmacol 2017;52:168–75. 28926773 10.1016/j.intimp.2017.09.008

[57] Griffiths JS , Camilli G, Kotowicz NK et al Role for IL-1 family cytokines in fungal infections. Front Microbiol 2021;12:633047. 33643264 10.3389/fmicb.2021.633047 PMC7902786

[58] Cenci E , Mencacci A, Casagrande A et al Impaired antifungal effector activity but not inflammatory cell recruitment in interleukin-6-deficient mice with invasive pulmonary aspergillosis. J Infect Dis 2001;184:610–7. 11494166 10.1086/322793

[59] Li XV , Leonardi I, Iliev ID. Gut mycobiota in immunity and inflammatory disease. Immunity 2019;50:1365–79. 31216461 10.1016/j.immuni.2019.05.023 PMC6585451

[60] Qiu X , Ma J, Jiao C et al Alterations in the mucosa-associated fungal microbiota in patients with ulcerative colitis. Oncotarget 2017;8:107577–88. 29296188 10.18632/oncotarget.22534 PMC5746090

[61] Roilides E , Anastasiou-Katsiardani A, Dimitriadou-Georgiadou A et al suppressive effects of interleukin-10 on human mononuclear phagocyte function against *Candida albicans* and Staphylococcus aureus. J Infect Dis 1998;178:1734–42. 9815227 10.1086/314479

[62] Monari C , Retini C, Palazzetti B et al Regulatory role of exogenous IL-10 in the development of immune response versus *Cryptococcus neoformans*. Clin Exp Immunol 1997;109:242–7. 9276518 10.1046/j.1365-2249.1997.4021303.x PMC1904742

[63] Roilides E , Dimitriadou A, Kadiltsoglou I et al IL-10 exerts suppressive and enhancing effects on antifungal activity of mononuclear phagocytes against *Aspergillus fumigatus*. J Immunol 1997;158:322–9. 8977206

[64] Montagnoli C , Bacci A, Bozza S et al B7/CD28-Dependent CD4+CD25+ regulatory T cells are essential components of the memory-protective immunity to *Candida albicans*. J Immunol 2002;169:6298–308. 12444136 10.4049/jimmunol.169.11.6298

[65] Mencacci A , Cenci E, Sero GD et al IL-10 is required for development of protective th1 responses in IL-12-deficient mice upon *Candida albicans* infection. J Immunol 1998;161:6228–37. 9834110

[66] Okada Y , Tsuzuki Y, Sugihara N et al Novel probiotic yeast from Miso promotes regulatory dendritic cell IL-10 production and attenuates DSS-induced colitis in mice. J Gastroenterol 2021;56:829–42. 34213612 10.1007/s00535-021-01804-0

[67] Rodríguez-Cerdeira C , Lopez-Bárcenas A, Sánchez-Blanco B et al The role of IL-33 in host response to *Candida albicans*. Sci World J 2014;2014;340690. 10.1155/2014/340690 PMC4130336 25136658

[68] Park SJ , Cho HR, Kwon B. Roles of IL-33 in resistance and tolerance to systemic *Candida albicans* infections. Immune Netw 2016;16:159–64. 27340384 10.4110/in.2016.16.3.159 PMC4917399

[69] Tran VG , Kim HJ, Kim J et al IL-33 enhances host tolerance to *Candida albicans* kidney infections through induction of IL-13 production by CD4+ T cells. J Immunol 2015;194:4871–9. 25847973 10.4049/jimmunol.1402986

[70] Schiering C , Krausgruber T, Chomka A et al The alarmin IL-33 promotes regulatory T-cell function in the intestine. Nature 2014;513:564–8. 25043027 10.1038/nature13577 PMC4339042

[71] De Luca A , Zelante T, D’Angelo C et al IL-22 defines a novel immune pathway of antifungal resistance. Mucosal Immunol 2010;3:361–73. 20445503 10.1038/mi.2010.22

[72] Conti HR , Shen F, Nayyar N et al Th17 cells and IL-17 receptor signaling are essential for mucosal host defense against oral candidiasis. J Exp Med 2009;206:299–311. 19204111 10.1084/jem.20081463 PMC2646568

[73] Thompson A , Orr SJ. Emerging IL-12 family cytokines in the fight against fungal infections. Cytokine 2018;111:398–407. 29793796 10.1016/j.cyto.2018.05.019 PMC6299256

[74] Marakalala MJ , Kerrigan AM, Brown GD. Dectin-1: a role in antifungal defense and consequences of genetic polymorphisms in humans. Mamm Genome 2011;22:55–65. 20700596 10.1007/s00335-010-9277-3 PMC3026934

[75] Huang J , Meng S, Hong S et al IL-17C is required for lethal inflammation during systemic fungal infection. Cell Mol Immunol 2016;13:474–83. 26166766 10.1038/cmi.2015.56 PMC4947823

[76] Bär E , Whitney PG, Moor K et al IL-17 regulates systemic fungal immunity by controlling the functional competence of NK cells. Immunity 2014;40:117–27. 24412614 10.1016/j.immuni.2013.12.002

[77] Verma A , Kroetz DN, Tweedle JL et al Type II cytokines impair host defense against an intracellular fungal pathogen by amplifying macrophage generation of IL-33. Mucosal Immunol 2015;8:380–9. 25118166 10.1038/mi.2014.75 PMC4326567

[78] Cenci E , Mencacci A, Del Sero G et al Interleukin-4 causes susceptibility to invasive pulmonary aspergillosis through suppression of protective type I responses. J Infect DIS 1999;180:1957–68. 10558953 10.1086/315142

[79] Müller U , Stenzel W, Köhler G et al IL-13 induces disease-promoting type 2 cytokines, alternatively activated macrophages and allergic inflammation during pulmonary infection of mice with *Cryptococcus neoformans*. J Immunol 2007;179:5367–77. 17911623 10.4049/jimmunol.179.8.5367

[80] Mencacci A , Del Sero G, Cenci E et al Endogenous interleukin 4 is required for development of protective CD4+ T helper type 1 cell responses to *Candida albicans*. J Exp Med 1998;187:307–17. 9449711 10.1084/jem.187.3.307 PMC2212115

[81] Katsifa H , Tsaparidou S, Diza E et al Effects of interleukin-13 on antifungal activity of human monocytes against *Candida albicans*. FEMS Immunol Med Microbiol 2001;31:211–7. 11720817 10.1111/j.1574-695X.2001.tb00522.x

[82] Antachopoulos C , Roilides E. Cytokines and fungal infections. Br J Haematol 2005;129:583–96. 15916680 10.1111/j.1365-2141.2005.05498.x

[83] Bacher P , Kniemeyer O, Schönbrunn A et al Antigen-specific expansion of human regulatory T cells as a major tolerance mechanism against mucosal fungi. Mucosal Immunol 2014;7:916–28. 24301658 10.1038/mi.2013.107

[84] Danese S , Rudziński J, Brandt W et al Tralokinumab for moderate-to-severe UC: a randomised, double-blind, placebo-controlled, phase IIa study. Gut 2015;64:243–9. 25304132 10.1136/gutjnl-2014-308004

[85] Walter Reinisch W , Panés J, Khurana S etal Anrukinzumab, an anti-interleukin 13 monoclonal antibody, in active UC: efficacy and safety from a phase IIa randomised multicentre study. Gut 2015;64:894–900. 25567115 10.1136/gutjnl-2014-308337

[86] Kasaian MT , Page KM, Fish S et al Therapeutic activity of an interleukin-4/interleukin-13 dual antagonist on oxazolone-induced colitis in mice. Immunology 2014;143:416–27. 24831554 10.1111/imm.12319 PMC4212955

[87] Fort MM , Lesley R, Davidson NJ et al IL-4 exacerbates disease in a Th1 cell transfer model of colitis. J Immunol 2001;166:2793–800. 11160346 10.4049/jimmunol.166.4.2793

[88] Jayme TS , Leung G, Wang A et al Human interleukin-4–treated regulatory macrophages promote epithelial wound healing and reduce colitis in a mouse model. Sci Adv 2020;6:eaba4376. 32548267 10.1126/sciadv.aba4376 PMC7274799

[89] Almradi A , Hanzel J, Sedano R et al Clinical trials of IL-12/IL-23 inhibitors in inflammatory bowel disease. BioDrugs 2020;34:713–21. 33105016 10.1007/s40259-020-00451-w

[90] McDonald BD , Dyer EC, Rubin DT. IL-23 monoclonal antibodies for IBD: so many, so different? J Crohn’s Colitis 2022;16:ii42–ii53. 35553664 10.1093/ecco-jcc/jjac038 PMC9097671

[91] Lee JS , Tato CM, Joyce-Shaikh B et al Interleukin-23-independent IL-17 production regulates intestinal epithelial permeability. Immunity 2015;43:727–38. 26431948 10.1016/j.immuni.2015.09.003 PMC6044435

[92] Maxwell JR , Zhang Y, Brown WA et al Differential roles for interleukin-23 and interleukin-17 in intestinal immunoregulation. Immunity 2015;43:739–50. 26431947 10.1016/j.immuni.2015.08.019

[93] Hueber W , Sands BE, Lewitzky S et al; for the Secukinumab in Crohn's Disease Study Group. Secukinumab, a human anti-IL-17A monoclonal antibody, for moderate to severe Crohn’s disease: unexpected results of a randomised, double-blind placebo-controlled trial. Gut 2012;61:1693–700. 22595313 10.1136/gutjnl-2011-301668 PMC4902107

[94] Fauny M , Moulin D, D’Amico F et al Paradoxical gastrointestinal effects of interleukin-17 blockers. Ann Rheum Dis 2020;79:1132–8. 32719044 10.1136/annrheumdis-2020-217927

[95] Deng Z , Wang S, Wu C et al IL-17 inhibitor-associated inflammatory bowel disease: a study based on literature and database analysis. Front Pharmacol 2023;14:1124628. 37033665 10.3389/fphar.2023.1124628 PMC10076642

[96] Eken A , Singh AK, Treuting PM et al IL-23R+ innate lymphoid cells induce colitis via interleukin-22-dependent mechanism. Mucosal Immunol 2014;7:143–54. 23715173 10.1038/mi.2013.33 PMC3834084

[97] Zhu Q , Korenfeld D, Suarez-Fueyo A et al Epithelial dysfunction is prevented by IL-22 treatment in a Citrobacter rodentium-induced colitis model that shares similarities with inflammatory bowel disease. Mucosal Immunol 2022;15:1338–49. 36372810 10.1038/s41385-022-00577-w

[98] Schreiber S , Fedorak RN, Nielsen OH et al Safety and efficacy of recombinant human interleukin 10 in chronic active Crohn’s disease. Gastroenterology 2000;119:1461–72. 11113067 10.1053/gast.2000.20196

[99] Wang X , Wong K, Ouyang W et al Targeting IL-10 family cytokines for the treatment of human diseases. Cold Spring Harb Perspect Biol 2019;11:a028548. 29038121 10.1101/cshperspect.a028548 PMC6360861

[100] Liso M , Verna G, Cavalcanti E et al Interleukin 1β blockade reduces intestinal inflammation in a murine model of tumor necrosis factor-independent ulcerative colitis. Cell Mol Gastroenterol Hepatol 2022;14:151–71. 35314399 10.1016/j.jcmgh.2022.03.003 PMC9120241

[101] Sivakumar PV. Interleukin 18 is a primary mediator of the inflammation associated with dextran sulphate sodium induced colitis: blocking interleukin 18 attenuates intestinal damage. Gut 2002;50:812–20. 12010883 10.1136/gut.50.6.812 PMC1773244

[102] Raine T , Vaja S, Subramanian S et al; The IASO Trial Investigators. OP33 Results of a randomised controlled trial to evaluate Interleukin 1 blockade with anakinra in patients with acute severe ulcerative colitis (IASO). J Crohn’s Colitis 2023;17:i43–i46.

[103] Danese S , Vermeire S, Hellstern P et al Randomised trial and open-label extension study of an anti-interleukin-6 antibody in Crohn’s disease (ANDANTE I and II). Gut 2019;68:40–8. 29247068 10.1136/gutjnl-2017-314562 PMC6839832

[104] Song Y , Yuan M, Xu Y et al Tackling inflammatory bowel diseases: targeting proinflammatory cytokines and lymphocyte homing. Pharmaceuticals 2022;15:1080. 36145301 10.3390/ph15091080 PMC9502105

[105] Chen W-Y , Tsai T-H, Yang J-L et al Therapeutic strategies for targeting IL-33/ST2 signalling for the treatment of inflammatory diseases. Cell Physiol Biochem 2018;49:349–58. 30138941 10.1159/000492885

[106] Wang Y , Mao Y, Zhang J et al IL-35 recombinant protein reverses inflammatory bowel disease and psoriasis through regulation of inflammatory cytokines and immune cells. J Cellular Molecular Medi 2018;22:1014–25. 10.1111/jcmm.13428 PMC5783847 29193791

[107] Vulliemoz M , Brand S, Juillerat P et al; on behalf of Swiss IBDnet, an official working group of the Swiss Society of Gastroenterology. TNF-alpha blockers in inflammatory bowel diseases: practical recommendations and a user’s guide: an update. Digestion 2020;101:16–26. 32739923 10.1159/000506898

[108] Hemperly A , Vande Casteele N. Clinical pharmacokinetics and pharmacodynamics of infliximab in the treatment of inflammatory bowel disease. Clin Pharmacokinet 2018;57:929–42. 29330783 10.1007/s40262-017-0627-0

[109] Iriarte A , Zaera C, Bachiller-Corral J et al Inflammatory bowel disease as a paradoxical effect of anti-TNF alpha therapy. Gastroenterol Hepatol 2017;40:117–21. 26993096 10.1016/j.gastrohep.2016.01.011

[110] Harris MS , Hartman D, Lemos BR et al AVX-470, an orally delivered anti-tumour necrosis factor antibody for treatment of active ulcerative colitis: results of a first-in-human trial. ECCOJC 2016;10:631–40. 10.1093/ecco-jcc/jjw036 26822613

[111] Schierova D , Roubalova R, Kolar M et al Fecal microbiome changes and specific anti-bacterial response in patients with IBD during anti-TNF therapy. Cells 2021;10:3188. 34831411 10.3390/cells10113188 PMC8617723

[112] Langer V , Britzen-Laurent N, Regensburger D et al P064 interferon-gamma induced vascular impairment contributes to the pathogenesis of inflammatory bowel diseases. Gastroenterology 2018;154:S34.

[113] Hommes DW. Fontolizumab, a humanised anti-interferon γ antibody, demonstrates safety and clinical activity in patients with moderate to severe Crohn’s disease. Gut 2005;55:1131–7. 10.1136/gut.2005.079392 PMC1856291 16507585

